# Effectiveness and Safety of a Shorter Treatment Regimen in a Setting with a High Burden of Multidrug-Resistant Tuberculosis

**DOI:** 10.3390/ijerph18084121

**Published:** 2021-04-13

**Authors:** Aleksandr Trubnikov, Arax Hovhannesyan, Kristina Akopyan, Ana Ciobanu, Dilbar Sadirova, Lola Kalandarova, Nargiza Parpieva, Jamshid Gadoev

**Affiliations:** 1 Abt Associates, Rashidov Street C-4, 20A, Yunosobod District, Tashkent 100093, Uzbekistan; 2 Republican Specialized Scientific Practical Medical Centre of Phthisiology and Pulmonology under Ministry of Health of the Republic of Uzbekistan, Sh. Alimov 1, Little Ring Road, Tashkent 100086, Uzbekistan; nargizaparpieva@gmail.com; 3 World Health Organization Regional Office for Europe, UN City, Marmorvej 51, DK-2100 Copenhagen, Denmark; dr.akopyan@gmail.com (K.A.); aciobanu@who.int (A.C.); 4 Tuberculosis Research and Prevention Center NGO, Yerevan 0023, Armenia; 5 Tashkent City Center of Phthisiology and Pulmonology, Lutfi Street 33/1, 7th District, Chilonzor District, Tashkent 100043, Uzbekistan; barchik.1976@mail.ru (D.S.); lola_nur69@mail.ru (L.K.); 6 World Health Organization Uzbekistan Country Office, 16, Tarobiy Street, Tashkent 100100, Uzbekistan; gadoevj@who.int

**Keywords:** shorter treatment regimen, drug adverse event, RR-TB treatment outcome, operational research, SORT IT, Uzbekistan

## Abstract

Treatment of drug-resistant tuberculosis is lengthy, insufficiently effective, and toxic. Since 2016, the World Health Organization has recommended shorter treatment regimens (STR). We assessed effectiveness and predictors of drug adverse events (DAE) among patients treated with STR. There were 95 consecutive rifampicin-resistant patients enrolled in STR in Tashkent between June 2018 and September 2019. Of these, 66.3% were successfully treated, 17.9% suffered failed treatment, 7.4% died, 5.3% were lost to follow-up and 3.2% were not evaluated. No recurrence was identified in 54 patients after 12 months of successful treatment completion. There were 47 reported DAE: the incidence rate was 6.15 DAE per 100 person-months-of-treatment. Any DAE was reported in 38 (40%) patients and grade 3/4 DAE were recorded in 21 (22.1%) patients. Median time to DAE was 101 (interquartile range 64–139) days. The most frequently encountered DAE were gastro-intestinal disorders, followed by hepatotoxicity and ototoxicity. The most commonly offending drug inducing DAE was protionamide. The dose was temporarily interrupted in 55.3% of DAE, reduced in 8.5% of DAE and permanently withdrawn in another 8.5% of DAE. HIV status was the only predictor associated with increased hazard of DAE. In Uzbekistan STR showed moderate effectiveness and safety, although treatment failure was high.

## 1. Introduction

Despite the availability of effective and affordable treatment for tuberculosis (TB) for several decades, the disease remains world’s most deadly infectious disease. In 2019 about 10 million people fell ill with TB and 1.4 million died [1]. Rifampicin-resistant/multidrug-resistant (RR/MDR TB) forms of tuberculosis make TB control even more challenging, because treatment is longer, more expensive, and is associated with worse outcomes compared to drug-sensitive forms. The long courses of treatment are often compounded by stigma, discrimination, social isolation, treatment fatigue and loss of job and income. Globally, only 57% of RR-TB patients enrolled for treatment were successfully treated in 2017.

Uzbekistan is one of 30 countries world-wide with a high burden of drug-resistant TB. Despite an accelerating decline of the TB burden in Uzbekistan, the per-capita rate of RR-TB diagnoses is still on the increase [2,3]. As of 2019, according to routine drug-resistance surveillance, the prevalence of RR/MDR TB among new patients in Uzbekistan was 12% and 22% among previously treated patients [1].

According to conventional second-line TB treatment guidelines, the duration of treatment could take up to 20 months or more [4]. This increases the likelihood of developing drug adverse events (DAE), which play an important role in discontinuation of treatment, resulting in high levels of treatment failure, poor adherence to treatment and ultimately losses to follow-up [5,6].

Between 2010 and 2016, a number of studies conducted in Bangladesh and several countries in Africa demonstrated that in selected patients the length of treatment of RR-TB patients could be reduced to 9 months [7,8,9,10,11]. This was a groundbreaking advance in TB management as, compared to conventional treatment, the shorter treatment regimen spares the RR-TB patients from long-lasting exposure to devastating side effects of TB drugs, reduces the total pill burden, and enables patients to more rapidly return to work and social activities. Based on promising results from these studies, the WHO in May 2016 included the STR among the recommended regimens for MDR-TB treatment under specific conditions [12].

Uzbekistan was the first and only country in the WHO European Region that has implemented a shorter treatment regimen under operational research conditions in Karakalpakstan since 2013, with the support of Medecins sans Frontieres (MSF), before WHO released its recommendations on shorter treatment regimens [13]. In mid-2018, Uzbekistan approved and introduced a short-course treatment regimen (STR) for RR-TB patients for routine programmatic implementation [14]. All other STR studies have been conducted in settings with a low RR-TB burden, which restricts the generalizability of those findings. In addition, there were debates on the appropriateness of the STR for the WHO European Region. Experts argued that in settings with a high prevalence of resistance to the drugs which were included in the regimen, only a minority of RR-TB patients would be suitable for STR [15]. Moreover, a recent qualitative study in Uzbekistan indicated that among health care providers there was doubt about the appropriateness and effectiveness of STR. Concerns about the toxicity of the STR were also raised [16].

Given the lack of information related to STR effectiveness and safety in settings with a high MDR-TB burden and considering that STR was recently introduced in Uzbekistan under routine programmatic conditions, our study aimed to (i) assess the treatment outcomes of patients treated with STR, (ii) assess the frequency, severity and time of onset of DAE after initiation of the STR and (iii) identify risk factors for DAE among the patients receiving STR in Tashkent between 2018 and 2019.

## 2. Materials and Methods

### 2.1. Study Design

We conducted a cohort study involving secondary analysis of routinely collected data.

### 2.2. Setting

Uzbekistan is a land-locked, middle income country situated in Central Asia with a population of about 33 million according to 2019 estimates. The capital city is Tashkent with a population of about 2.5 million. Management of TB is free of charge and is performed exclusively by health facilities under the public sector.

According to routine surveillance data, in 2019, about 75% of notified new and relapse TB patients were tested using Xpert MTB/RIF. External quality assurance is implemented by the Supra National Reference Laboratories in Borstel and Gauting, Germany.

The treatment success rate of RR-TB patients in Uzbekistan increased from 52.5% in 2013 to 60.6% in 2017 [3]. Starting from June 2018, the National Tuberculosis program introduced the STR in Tashkent according to the clinical protocol for the treatment of drug-resistant forms of tuberculosis with short-course treatment regimens [14]. According to routine surveillance reports in 2018 of 2238 notified RR-TB patients, 137 (6.1%) were enrolled into STR. In 2019 of 2060 RR-TB cases, 157 (7.6%) received STR treatment in Uzbekistan.

To address and monitor the increased risk of drug toxicity with the new treatment regimen and new and repurposed TB drugs, an interim package of active pharmacovigilance was adopted in the tuberculosis control service of Uzbekistan in-line with international recommendations [17,18]. According to this package of pharmacovigilance, the occurrence of serious AE and AE of special interest are recorded and reported in the National Tuberculosis Program (NTP).

### 2.3. Study Population

All consecutive RR-TB patients who started STR in six selected facilities in Tashkent city from June 2018 to September 2019 were included in the study. RR-TB patients were eligible for STR if they were affected by *M. tuberculosis* strains that were sensitive to both fluroquinolones and second-line injectable drugs, if they had not been previously treated for more than one month with second-line drugs and did not have extensive forms of disease. Children aged <15 years, pregnant women, patients with resistance to both kanamycin and capreomycin, as well as those with corrected QT interval prolongation beyond 500 ms also were deemed not eligible for STR.

### 2.4. Diagnosis, Treatment and Monitoring

The diagnosis of TB in Uzbekistan is established by direct sputum smear microscopy and Xpert MTB/RIF, supported by chest radiography and culture. Patients diagnosed with rifampicin resistance are then tested for resistance for fluoroquinolones and second-line injectables using the line-probe assay (Genotype^®^ MTBDRsl assay, Hain Lifescience, Nehren, Germany) and/or phenotypic drug sensitivity test (DST) either on liquid or solid media. Decisions about enrollment into DR-TB treatment, the choice of regimen and further modifications are undertaken by the DR-TB consilium.

The STR regimen adopted in Uzbekistan includes moxifloxacin (Mfx), capreomycin (Cm), clofazimine (Cfz), protionamide (Pto), pyrazinamide (Z), ethambutol (E) and high-dose isoniazid (H) administered daily during the intensive phase of treatment and Mfx Cfz Pto Z E during the continuation phase of treatment. Patients start treatment with capreomycin but may be switched to kanamycin if DST indicates susceptibility to kanamycin. The duration of the intensive phase of treatment is four months, but this can be extended up to six months if sputum and/or culture is still positive at the fourth month [14,19]. The duration of the continuation phase is 5 months (Table S1).

Typically, all patients with RR-TB are hospitalized during the first two months of the treatment. Then, the treatment is continued under direct observation of an outpatient health facility near to the area of residence. Treatment progress is monitored by sputum smear microscopy and culture testing on liquid media on a monthly basis. Clinical evaluation, full blood count, liver function tests, electrocardiography (ECG) and testing of blood for potassium is conducted weekly during the first month of treatment and then monthly until the end of the treatment. Audiograms (during the intensive phase of treatment), visual acuity, blood sugar, creatinine tests are performed on a monthly basis, and thyroid stimulating hormone is measured before the start of the treatment and at month 6 and month 12. Psychological evaluation is conducted twice during the first month, and then monthly [20]. All health care providers involved in the management of DR-TB are trained on the new treatment guidelines and in the identification, recording, reporting and management of DAE.

### 2.5. Data Collection

During the intensive phase of treatment, patients are kept under daily supervision either at in-patient or outpatient settings. All clinical and demographic data are recorded in individual medical history and standard treatment cards. In the case of occurrence of DAE, TB physicians make corresponding records in the medical history and complete the appropriate reporting forms. For the study, we collected data directly from clinical records in the selected health facilities and these data were directly entered into standard electronic records developed using the EpiData application (version 3.1 EpiData Association, Odense, Denmark). Inconsistencies were resolved by analyzing the source documents.

Information extracted from medical records included patient identification number, date of treatment initiation, date of birth, sex, category, site of disease, radiological diagnosis, comorbidities, weight and height measurements at treatment initiation, HIV status, anti-retroviral treatment, treatment outcome, date of treatment outcome, as well as the characteristics of the DAEs, including type, date of onset, severity grade, management, the suspected drug and the outcome.

### 2.6. Definitions

DAE are defined as noxious and unintended effects of TB medicine administered at the appropriate dosages, which are recorded in the clinical form by treating physicians [17,18]. Severity grade of DAE was assigned according to the national manual on pharmacovigilance for TB control, which follows the National Institute of Allergy and Infectious Diseases, Division of AIDS (DAIDS) system [21].

The follow-up period was defined as the time from the date of the initiation of TB treatment with second-line drugs to the date of the establishment of the treatment outcome. The definition of treatment outcomes is shown in Table S2.

### 2.7. Analysis and Statistics

The participants’ baseline and follow-up characteristics and treatment outcomes were described using frequencies and percentages for categorical variables, and medians and interquartile ranges (IQR) for continuous variables. The age variable was converted into two categories split at the age of 40 years. Nutrition status was dichotomized by the body mass index (BMI) as “below 18.5 kg/m^2^ “, and “18.5 kg/m^2^ and over” as per WHO classification [22].

The incidence rate of DAE was expressed as the total number of adverse drug events divided by the overall follow-up time per 100 person-months. Variables associated with DAE were evaluated by using Andersen–Gill Cox survival analysis with subjects censored at the death of the patient, treatment discontinuation, treatment failure and end of the treatment. This approach allows for the accounting of multiple events per patient over the entire follow-up period [23]. We calculated the crude hazard ratio of DAE across the categories of covariates assessed. Factors associated with DAE at the 0.2 significance level in univariable analysis were included into a multivariable Cox model. Age and sex a priori were included in the final model, as well as those variables which reached a significance level of 0.05 as predictors of DAE. Two-sided *p* values in regression analyses were derived from likelihood ratio tests. Departure from the proportional hazard assumption was tested by incorporating interaction terms between the time period of follow-up and the risk factors in the final multivariable model. In addition, the global test of proportionality using Schoenfeld residuals was also used in the assessment. All statistical tests were two-tailed. Statistical analysis was completed using Intercooled Stata software version 15 (Stata Corp. College Station, TX, USA).

We used the RawGraphs web-application to construct alluvial plots to visualize the spectrum of DAE [24].

## 3. Results

### 3.1. Baseline Charachteristics and Cohort Description

The study population consisted of 95 laboratory-confirmed RR/MDR-TB patients. Mean (SD) age of study participants was 45.6 (16.1) years ranging from 17.8 to 82.3 years at the time of enrollment. Of all participants, 67 (70.5%) were male and 46 (48.4%) were categorized as new cases. All patients had documented HIV test results, and 11 (11.6%) were co-infected with HIV. Of those that tested HIV positive, only six (54.5%) were on anti-retroviral treatment (ART). Two (2.1%) patients had extra pulmonary localization (tuberculous pleurisy). Among 93 patients with pulmonary localization, 11 (11.6%) had cavities and six (6.3%) had disseminated pulmonary TB. Diabetes mellitus was diagnosed in 18 (28.1%) patients. Of 89 patients with anthropometric data, 21 (26.9%) were underweight (BMI < 18.5 kg/m^2^). Other demographic and clinical characteristics of the study population are summarized in Table 1.

### 3.2. Treatment Outcome

Of 95 patients enrolled into treatment, 63 (66.3%) were successfully treated, 17 (17.9%) suffered failed treatment, 7 (7.4%) died, 5 (5.3%) were lost to follow-up and three (3.2%) were not evaluated. No cases of recurrent TB were identified among 54 successfully treated patients assessed after 12 months of treatment completion.

Median duration of follow-up time of all 95 patients was 9.0 months (IQR 5.4–9.7). Thirty-two patients were censored due to death, lost to follow-up, treatment failure and unknown treatment outcome over a median of 4.0 (IQR = 2.3–6.0) months. Median follow-up time of 63 patients with successful treatment outcome was 9.4 (IQR = 9.1–10.3) months. In seven patients, the treatment duration based on clinical and radiological findings was extended beyond 12 months with a maximum of 15.5 months.

### 3.3. Characteristics of Drug Adverse Events

**Figure 1:**
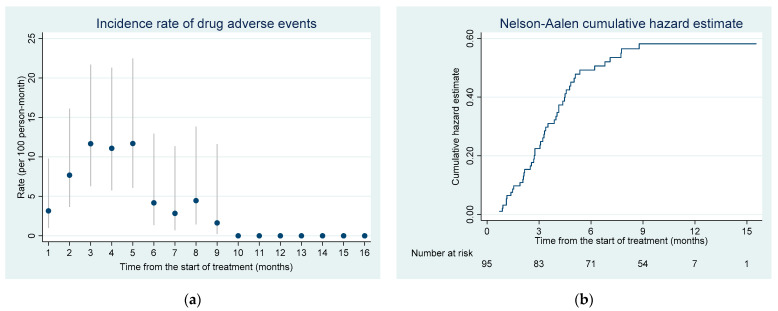
(**a**) Incidence rate of drug adverse events over the course of treatment; vertical lines indicate the 95% confidence interval; the incidence rate was estimated for monthly time intervals, for example the rate at month 3 indicates the period above 2 months and less or equal to 3 months; (**b**) cumulative hazard of drug adverse events since the start of the treatment among RR/MDR-TB patients enrolled in shorter treatment in Tashkent, Uzbekistan.

Among 95 patients, 38 (40.0%) experienced at least one DAE over the course of their treatment: 29 had one occurrence of DAE, and nine had two DAE, giving a total of 47 DAE. All 47 DAE occurred over 764 person-months follow up, giving a cumulative incidence rate of 6.15 (95% CI 4.62–8.18) DAE per 100 person-months. The probability of DAE gradually increased after the start of treatment reaching the highest levels between the 3rd to 5th months of the treatment and then decreased with some variation. The latest DAE was recorded as late as the 9th month after the initiation of STR treatment (Figure 1).

The majority (59.6%) of the DAE were graded 3 or 4 in terms of severity while DAE of grade 1 and grade 2 accounted for 6.4% and 34.0% of total events, respectively (Table 2). Grade 3 or 4 DAEs were recorded in 21 (22.1%) patients. Of those, seven patients each experienced two DAE of grade 3 or grade 4. The overall rate DAE of grade 3 or grade 4 was 3.66/100 persons-month.

The most frequently encountered DAE were gastro-intestinal (nausea, vomiting) disorders, with an incidence of 1.57 per 100 person-month (12 events), followed by hepatotoxicity with an incidence of 1.05 event per 100 person-month (8 events), ototoxicity (3 events of hearing loss, 3 events of tinnitus and one event of ataxia) and dermatologic disorders with an incidence of 0.92 person-months (7 events each).

**Figure 2:**
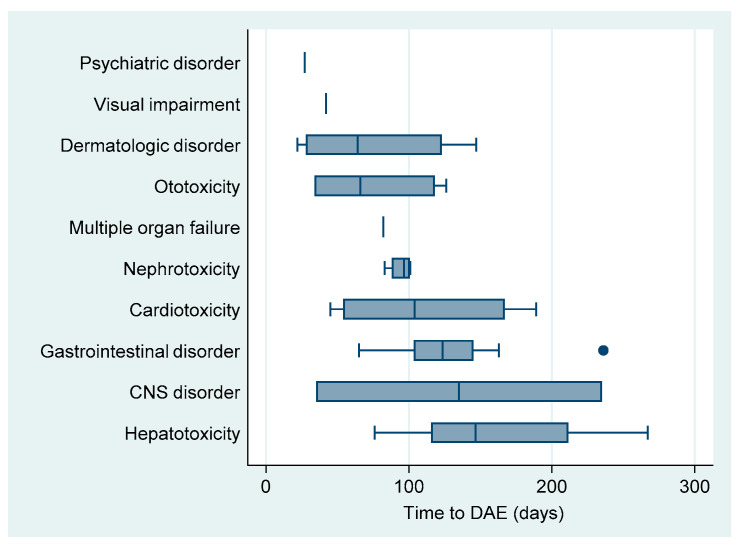
Boxplot of median time until onset of drug adverse event in patients on shorter treatment regimens in RR/MDR-TB patients in Uzbekistan.

The median time to DAE was 101 (IQR: 62–139) days. By the onset of occurrence, psychiatric disorders (acute psychosis) and visual impairment (optic neuritis) were the earliest DAE, although they were recorded only once among our study population. The latest occurring DAE was hepatotoxicity with a median time of onset of 146 days after treatment initiation (Table 2, Figure 2).

The most frequently suspected agent was protionamide, which was responsible for one in three DAE followed by second-line injectables (10 events) and clofazimine (6 events). As four patients developed a recurrent DAE from the same agent, we calculated toxicity of each drug in the regimen as a proportion of patients experiencing DAE. Altogether, 15.8% developed AEs from protionamide. Second line injectables (kanamycin or capreomycin) were the second most commonly reported drugs with high toxicity (10.5% of exposed). The toxicities of Cfz, Z and E were comparable (4.2% each) (Table 3).

**Figure 3:**
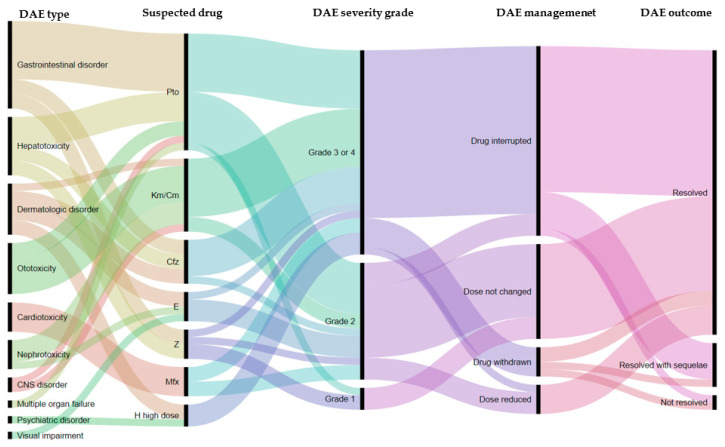
Alluvial plot illustrating the relationship between the types of adverse event, the suspected drug, the severity grade, the management and outcome of the adverse event among RR/MDR-TB patients on the shorter treatment regimen in Tashkent, Uzbekistan.

To manage DAE in four (8.5%) instances, a reduction in dosage was the effective solution, in 26 (55.3%) instances the suspected drug(s) were interrupted temporally, while in four (8.5%) instances the suspected drug(s) were permanently discontinued. Drugs that were permanently discontinued were kanamycin, protionamide and pyrazinamide. In the majority of cases (80.1%) DAE were resolved, while in six instances (12.8%) DAE were resolved with sequelae and in two instances DAE were not resolved (4.3%). More specifically, the treatment of patients was interrupted, and patients were assigned a treatment outcome of failure—both of these patients died soon after stopping TB treatment. The spectrum of DAE, the suspected drugs, the management and outcome are shown in Table 4 and Figure 3.

### 3.4. Predictors of Drug Adverse Events

HIV positive patients had an 81% increased risk of developing DAE compared to those who were HIV negative (adjusted hazard ratio (aHR) = 1.81; 95%CI (1.07–3.06)). This association became stronger when patients who were not receiving ART were excluded from the analysis (aHR = 2.08, 95%CI 1.26–3.44, *p* = 0.004) and disappeared when HIV positive TB patients receiving ART were excluded from the analysis (aHR = 1.63, 95%CI (0.79–3.34), *p* = 0.182).

Age, sex, previous history of treatment, body-mass index, alcohol use, year of registration, as well as comorbidities were not associated with DAE (Table 5).

## 4. Discussion

We assessed the treatment outcome and spectrum of DAE among RR/MDR-TB patients enrolled in STR treatment in Uzbekistan. Of all participants 66.3% (95% CI 55.9–75.7%) were successfully treated. This is comparable to the MSF-supported project results implemented in Karakalpakstan Autonomous Republic showing 71.9% treatment success among 128 RR-TB patients enrolled in STR between 2013 and 2015 [13]. The effectiveness of STR in our study population is lower compared to 78.8% the success rate achieved with the STR in the STREAM trial [25]. Most of the STR studies conducted in Africa and Asia have shown impressive treatment success ranging between 80.2% and 95.5% [8,9,11,26,27]. Treatment effectiveness of STR was comparatively worse in Swaziland (70.8%) [10] and Guinea (74.0%) [28], but nevertheless much higher than treatment effectiveness of the longer regimen reported in their countries. In our study population treatment failure was the predominant reason of unfavorable treatment outcome, accounting for 17.9% of all patients. This is much higher than the reported 9.7% treatment failure for the longer MDR-TB regimen (excluding XDR-TB) in Uzbekistan in 2017. Four out of 17 cases of treatment failure were related to regimen intolerability. A limitation of this analysis is that we did not have information on drug susceptibility status of first and second-line drugs at baseline or on acquired drug resistance, and this may have accounted for the observed higher rate of treatment failure. There might be several explanations for the observed high rate of treatment failure.

First, the STR regimen is administered irrespective of the resistance pattern of companion drugs. A representative nationwide study conducted earlier indicated that more than two-thirds of MDR-TB cases in Uzbekistan were resistant to ethambutol [29], indicating a high likelihood of resistance to companion drugs in our study population, Thus, there is a high likelihood that many patients in our study population had additional resistance to companion drugs which might have rendered the STR regimen ineffective. Although the nine-country study in Africa demonstrated that there was no association between pyrazinamide and ethambutol resistance and treatment failure [11], a more recent individual meta-analysis, which included a large number of observations of treatment failure, provided sound evidence that, in the presence of resistance to pyrazinamide, ethambutol, or prothionamide/ethionamide, the shorter regimen was associated with more failure and relapse compared to the longer regimen [30]. The study conducted in Karakalpakstan also demonstrated that resistance to pyrazinamide and ethambutol was strongly associated with treatment failure [13].

Second, unlike the other countries showing high effectiveness of STR, resistance to fluoroquinolones and second-line injectables, which are the pillars of the STR, is substantially higher in Uzbekistan. Thus, according to the routine drug-resistance surveillance of 2019 in Uzbekistan, 44.8% of RR/MDR-TB patients who were tested had resistance to fluoroquinolones and 34.8% had extensively drug-resistant tuberculosis (XDR-TB). Although patients are tested for second line DST, this high prevalence of resistance to second line drugs in the population upsets the negative predictive values of DST (i.e., the probability of classifying a result as a false negative), which in turn increases the risk of enrolling bacteriologically non-eligible patients into STR.

Third, some of the studies in Africa included gatifloxacin in their regimen which has been shown to be superior to moxifloxacin or levofloxacin in STR in terms of bacteriological outcomes [31], while other studies have used higher dose of fluoroquinolones [7,25]. The Uzbekistan standard STR regimen included moxifloxacin at 400 mg.

The treatment success rates achieved using STR in our study population is comparable to the treatment success rates of RR-TB patients treated with conventional regimens in Uzbekistan (60.6%). However, this comparison should be made with caution due to possible selection bias as the patients with second-line drug-resistance and extensive forms of disease are not eligible for STR. In addition, seven of the patients received TB treatment beyond 12 months duration, which is not in-line with the short course treatment protocol.

In our study, grade 3 or 4 DAE occurred in 22.1% (95%CI 14.2–31.9%) of participants. This is much higher compared to STR observational studies conducted in nine countries in Africa (10.7%) [9,11], Niger (6.6%) [26] and Burundi (4%) [32], but much lower compared to the 48.2% of grade 3–4 AE reported in the STR arm in the STREAM trial [25]. Our results are consistent with previous publications showing 21.9% grade 3 or 4 AE among RR-TB patients enrolled in STR in Karakalpakstan [13].

In our study 40% (95%CI 30.1–50.6%) of patients developed at least one DAE. This is lower compared to a study conducted in Uzbekistan by Kalandarova et al. among MDR/TB patients receiving conventional MDR-TB treatment in Tashkent showing that 55% of patients receiving ambulatory care and 86% of those receiving hospital-based care developed DAE using the conventional RR-TB regimen [33]. The proportion of patients developing at least one AE in STR studies conducted elsewhere has ranged from 50 to 89% [9,11,26,32,34]. Differences may be explained by differences in treatment regimens, AE definitions and the capacity and available resources for monitoring treatment. The comparatively lower frequency of mild and moderate adverse events in our study population may be attributable to a number of factors. We speculated that, at an early stage of introduction of pharmacovigilance, the physicians would be inclined to report all DAE, while over the time with the accumulation of experience, care providers would be less likely to record mild and moderate DAE, especially those not requiring the administration of auxiliary medicines or regimen modification.

In our study population the risk of development of DAE was highest between month 3 and month 5 of STR treatment. Such a pattern might be explained in that some DAE, such as ototoxicity, commonly are observed in patients receiving large cumulative doses of second-line injectable drug [4], while sharp decline between month 5 and 6 reflects the phasing out of the intensive phase of treatment. A similar pattern was shown among hospitalized RR-TB patients receiving conventional individualized treatment in Tashkent [33], but in other STR studies the median time to AE was reported earlier, usually during the first two months of treatment [11,32,34].

We found that patients testing positive for HIV had an increased risk of DAE. Increased likelihood of DAE among HIV positive patients might be explained by overlapping and additive drug toxicity of ART and second line TB drugs, and this has been described elsewhere [35]. As our cohort included both patients who received ART and were ART naïve, we explored the effects of ART on this association. The exclusion of ART in naïve HIV positive patients using the multivariate Cox model increased the strength of association between HIV and DAE, while the association completely disappeared when the analysis was limited to ART naïve HIV positive patients.

Although patients of older age, those under-nourished and those with co-morbid conditions had an increased hazard of DAE, these associations did not reach statistical significance in our study population, most likely to due to small sample sizes. This is in contrast to other studies conducted among RR/MDR-TB patients on the longer treatment regimen [36,37].

Our study was limited by its observational nature and small sample size. Another limitation is that we were unable to report data on drug resistance before and during the treatment or assess magnitude or possible impact of baseline and acquired drug resistance on treatment outcome. Additionally, in 12 patients with successful treatment outcomes, results of one-year follow-up post-TB treatment were not available.

An important strength of our study is that we used Andersen–Gill proportional risk modeling which allows for the accounting of multiple events. Only a few previous studies have used this approach to explore the frequency and pattern of AEs among patients receiving second-line drugs [38].

Treatment of drug resistant TB in high MDR-burden settings remains a serious challenge, even with shorter courses of treatment showing excellent results in countries of Africa and Asia. High rates of treatment failure are concerning. In 2021 Uzbekistan will introduce an all-oral short treatment regimen under operational research conditions, which will include bedaquiline, delamanid and linezolid. This regimen has already demonstrated promising results in Georgia [39], a country sharing a similar epi-profile with Uzbekistan in terms of RR-TB burden and prevalence of second-line drug resistance. This regimen will resolve the concerns of the STR regimen by improving the number of effective drugs in the regimen, reducing the pill burden and phasing-out second-line injectables and protionamide, and this will provide the potential to improve both treatment effectiveness and tolerability.

## 5. Conclusions

In Uzbekistan, the STR showed moderate effectiveness and moderate tolerability. However, treatment failure was frequent. This finding indicates the need for much rigorous identification and documentation of the drug susceptibility results of all drugs in the regimen among the patients enrolled in the STR. Susceptibility to companion drugs should be accounted in the decision of treatment regimen. Further prospective studies are needed to understand the impact and risk factors of acquired resistance to second-line drugs among the patients receiving RR-TB treatment.

This is one of the few studies demonstrating STR outcomes in a high RR-TB and high second-ling drug resistance burden setting, and as such this adds important information to the global body of knowledge on the extent of generalizability of the short course treatment regimen.

## Figures and Tables

**Table 1:** Clinical and demographic characteristics of rifampicin-resistant tuberculosis (RR-TB) patients enrolled into shorter treatment regimens (STR) in Tashkent city (2018–2019), (*n* = 95).

Characteristics	*N*	%
**Sex**		
Male	67	70.5
Female	28	29.5
**Age**		
<40 years	33	34.7
≥40 years	62	65.3
**Category**		
New	46	48.4
Retreated	49	51.6
**HIV status**		
Negative	84	88.4
Positive	11	11.6
**Antiretroviral treatment (among HIV pos)**		
Yes	6	54.5
No	5	45.5
**Body-mass index**		
≥18.5 kg/m^2^	58	74.4
<18.5 km/m^2^	21	26.9
missing	16	
**Clinical presentation**		
Disseminated PTB	6	6.3
Focal PTB	11	11.6
Infiltrative PTB	62	65.3
Pulmonary tuberculoma	3	3.2
Cavernous PTB	4	4.2
Fibrous-cavernous PTB	7	7.4
Tuberculous pleurisy	2	2.1
**Any complication**		
No	72	75.8
Yes	23	24.2
**Any comorbidity**		
No	39	41.1
Yes	56	58.9
**Diabetes**		
Yes	18	28.1
No	46	71.9
Missing	31	
**Hepatitis**		
Yes	19	29.7
No	45	70.3
Missing	31	
**Anemia**		
Yes	33	51.6
No	31	48.4
Missing	31	
**Year of treatment start**		
2018	48	50.5
2019	47	49.5

PTB: pulmonary TB; STR: Shorter treatment regimen.

**Table 2:** Frequency, severity, time of onset of drug adverse events among 95 RR-TB patients enrolled in shorter treatment regimen.

Drug Adverse Event Type	Total		Grade			Rate Per 100 PM	Median Time to DAE ^1^	(IQR) ^1^	(Range) ^1^
	*N*	(% of all)	I	II	III–IV				
Cardiotoxicity	4	(8.5)	0	2	2	0.524	104	(54–167)	(45–189)
Central nervous system disorder	2	(4.3)	0	0	2	0.262	135	(35–235)	(35–235)
Dermatologic disorder	7	(14.9)	0	3	4	0.916	64	(28–123)	(22–147)
Gastrointestinal disorder	12	(25.5)	2	4	6	1.571	123	(103–145)	(65–236)
Hepatotoxicity	8	(17.0)	1	4	3	1.047	146	(115–211)	(76–267)
Multiple organ failure	1	(2.1)	0	0	1	0.131	82		
Nephrotoxicity	4	(8.5)	0	2	2	0.524	96	(88–100)	(83–101)
Ototoxicity	7	(14.9)	0	1	6	0.916	66	(34–118)	(34–126)
Psychiatric disorder	1	(2.1)	0	0	1	0.131	27		
Visual impairment	1	(2.1)	0	0	1	0.131	42		
Total	47	(100.0)	3	16	28	6.152	101	(64–139)	(22–267)

^1^ Expressed in days; DAE: drug adverse event; PM: person/month; IQR: interquartile range.

**Table 3:** Suspected drugs for drug adverse events in 95 RR-TB patients enrolled in shorter treatment regimens.

Suspected Drug	Number DAEs	Number Patients with DAE	Toxicity (%)
Moxifloxacin	4	3	(3.2)
Kanamycin/capreomycin	10	10	(10.5)
Clofazimine	6	4	(4.2)
Protionamide	16	15	(15.8)
Pyrazinamide	4	4	(4.2)
Ethambutol	4	4	(4.2)
High dose isoniazid	3	3	(3.2)

**Table 4:** Management and outcome of DAEs among RR-TB patients enrolled in shorter treatment regimens in Tashkent.

Type of DAE	*N*	Management of DAE				Outcome of DAE		
		Dose Not Changed	Dose Reduced	Drug Interrupted	Drug(s) Withdrawal	Resolved	Resolved with Sequelae	Not Resolved
Cardiotoxicity	4	1	1	2	0	4	0	0
CNS disorder	2	0	0	2	0	2	0	0
Dermatologic disorder	7	0	1	6	0	7	0	0
Gastro-intestinal disorder	12	5	1	5	1	12	0	0
Hepatotoxicity	8	4	1	3	0	8	0	0
Multiple organ failure	1	0	0	0	1	0	1	0
Nephrotoxicity	4	2	0	1	1	4	0	0
Ototoxicity	7	1	0	5	1	1	4	2
Psychiatric disorder	1	0	0	1	0	1	0	0
Visual impairment	1	0	0	1	0	0	1	0
Total	47	13	4	26	4	39	6	2

DAE: drug adverse event; CNS: Central nervous system.

**Table 5:** Results of Andersen–Gill Cox regression of the patients factor associated with time to adverse event among 95 RR-TB patients receiving a short course treatment regimen in Tashkent, Uzbekistan (2018–2019).

Characteristics	*N*	DAE (*n*)	Follow-Up Time (100 * Month)	Rate (100 p/m)	Univariable			Multivariable		
					HR	95%CI	*p* Value	aHR	95%CI	LRT *p* Value
**Sex**										
Male	67	28	5.253	5.33	0.69	(0.40–1.20)	0.187	0.64	(0.37–1.09)	0.102
Female	28	19	2.392	7.94	1.00			1.00		
**Age group**										
<40 years	33	15	2.890	5.19	1.00			1.00		
≥40 years	62	32	4.755	6.73	1.24	(0.72–2.13)	0.445	1.21	(0.71–2.08)	0.483
**TB treatment history**										
New	46	22	3.814	5.77	1.00					
Retreated	49	25	3.831	6.53	1.15	(0.68–1.99)	0.592			
**HIV**										
Negative	84	38	6.600	5.76	1.00			1.00		
Positive	11	9	1.045	8.61	1.79	(1.05–3.07)	0.032	1.81	(1.07–3.06)	0.026
**ART (among HIV pos)**										
yes	6	5	0.671	7.45						
no	5	4	0.374	10.70						
**Body-mass index**										
≥18.5 kg/m^2^	58	24	4.795	5.01	1.00					
<18.5 km/m^2^	21	15	1.586	9.46	1.82	(0.98–3.37)	0.057			
missing	16									
**Any complication**										
No	72	39	6.023	6.48	1.00					
Yes	23	8	1.543	5.18	0.75	(0.33–1.68)	0.481			
**Any comorbidity**										
No	39	14	3.061	4.57	1.00					
Yes	56	33	4.484	7.36	1.61	(0.90–2.88)	0.111			
**Diabetes**										
Yes	18	10	1.400	7.14	1.03	(0.50–2.14)	0.921			
No	46	25	3.718	6.72	1.00					
Missing	31									
**Hepatitis**										
Yes	19	11	1.546	7.12	1.11	(0.54–2.31)	0.771			
No	45	24	3.573	6.72	1					
Missing	31									
**Anemia**										
Yes	33	13	2.583	5.03	0.62	(0.31–1.22)	0.615			
No	31	22	2.535	8.68	1					
Missing	31									
**Alcohol use**										
Yes	18	8	1.326	6.03	0.96	(0.46–2.00)	0.919			
No	71	37	5.785	6.40	1					
Missing	6									
**Year of registration**										
2018	48	20	3.961	5.05	1					
2019	47	27	3.763	7.17	1.36	(0.78–2.35)	0.276			

DAE: Drug adverse event; ART: Antiretroviral therapy; CI: Confidence Interval; p/m: person-month; HR: Hazard ratio; aHR: Adjusted hazard ratio; LRT: Likelihood ratio test.

## Data Availability

The data that support the findings of this study are available from the corresponding author, (A.H.), upon reasonable request.

## Supplementary Materials

The following are available online at https://www.mdpi.com/article/10.3390/ijerph18084121/s1, Table S1: Definition of treatment outcome, Table S2: Composition and dosing of standard STR regimen in Uzbekistan.

## ©World Health Organization 2021

All rights reserved. The WHO Regional Office for Europe has granted the Publisher permission for the reproduction of this article.

## Open Access Statement

In accordance with WHO’s open-access publication policy for all work funded by WHO or authored/co-authored by WHO staff members, the WHO retains the copyright of this publication through a Creative Commons Attribution IGO license (http://creativecommons.org/licenses/by/3.0/igo/legalcode (accessed on 12 March 2021)) which permits unrestricted use, distribution and reproduction in any medium provided the original work is properly cited.
