# Supplementary material for: Effectiveness and Safety of a Shorter Treatment Regimen in a Setting with a High Burden of Multidrug-Resistant Tuberculosis
Source: Int J Environ Res Public Health. 2021 Apr 13;18(8):4121. doi: 10.3390/ijerph18084121 (PMC8069801; doi:10.3390/ijerph18084121)
Supplement: Supplementary file 1 [file ijerph-18-04121-s001.pdf]

Table S1: Composition and dosing of standard STR regimen in Uzbekistan

Drug	Daily dose	Weight range				
		30-35kg	36-45kg	46-55kg	56-70kg	>70kg
**High dose isoniazid (H)**	**10mg/kg once daily**	300mg	400mg	500mg	600mg	600mg
Pyrazinamide (Z)	20-30 mg/kg once daily	800mg	1000mg	1200mg	1600mg	2000mg
Ethambutol (E)	15-25 mg/kg once daily	600mg	800mg	1000mg	1200mg	1200mg
Moxifloxacin (Mfx)	400 mg once daily	400mg	400mg	400mg	400mg	400mg
Protionamide (Pto)	500-750 mg once daily	500mg	500mg	500mg	750mg	750mg
Clofazimine (Cfz)	200-300mg daily (during the first two months of treatment) then dose reduced up to 100mg daily (alternatively 100mg daily)					

		30-33kg	34-40kg	41-45kg	46-50kg	51-70kg	>70kg
Capreomycin (Cm) or	15-20mg/kg once daily	500mg	600mg	750mg	800mg	1000mg	1000mg
Kanamycin (Km)	15-20mg/kg once daily	500mg	625mg	750mg	875mg	1000mg	1000mg

Table S2. Definition of treatment outcome.

**Cure**	**A patient with bacteriologically confirmed RR-TB who has completed treatment without evidence of failure AND at least three or more consecutive cultures taken at least 30 days apart are negative at the end of treatment.**
Treatment completion	Treatment completed without evidence of failure BUT no record that three or more consecutive cultures taken at least 30 days apart are negative at the end of treatment
Treatment success	"Cured" or "Treatment completed" (=treatment success) without recurrence over the 12-month follow-up period.
Failure	Patient fails to show culture negative by the end of month 6 of prolonged intensive phase; or Culture reversion during the continuation phase; or amplification of resistance with either ofloxacin, capreomycin, or kanamycin resistance; or adverse drug reactions (leading to permanent termination or the change of at least two anti-TB drugs in the regimen)
Died	A patient who dies for any reason during the course of treatment
Lost to follow-up	Interruption of treatment for ≥ 2 consecutive months without medical approval and not meeting criteria for failure
Not evaluated	A patient for whom no treatment outcome is assigned (this includes patients "transferred out" to another treatment unit and whose treatment outcome is unknown)
Reoccurrence	An episode of TB which occurred during the 12-month follow-up period after cure or treatment completion with two consecutive positive cultures, or one positive culture with clinical signs and symptoms or radiographic deterioration.
